# IgE-Related Chronic Diseases and Anti-IgE-Based Treatments

**DOI:** 10.1155/2016/8163803

**Published:** 2016-12-21

**Authors:** Arnau Navinés-Ferrer, Eva Serrano-Candelas, Gustavo-J Molina-Molina, Margarita Martín

**Affiliations:** Biochemistry Unit, Biomedicine Department, Faculty of Medicine, University of Barcelona, Casanova 143, 08036 Barcelona, Spain

## Abstract

IgE is an immunoglobulin that plays a central role in acute allergic reactions and chronic inflammatory allergic diseases. The development of a drug able to neutralize this antibody represents a breakthrough in the treatment of inflammatory pathologies with a probable allergic basis. This review focuses on IgE-related chronic diseases, such as allergic asthma and chronic urticaria (CU), and on the role of the anti-IgE monoclonal antibody, omalizumab, in their treatment. We also assess the off-label use of omalizumab for other pathologies associated with IgE and report the latest findings concerning this drug and other new related drugs. To date, omalizumab has only been approved for severe allergic asthma and unresponsive chronic urticaria treatments. In allergic asthma, omalizumab has demonstrated its efficacy in reducing the dose of inhaled corticosteroids required by patients, decreasing the number of asthma exacerbations, and limiting the effect on airway remodeling. In CU, omalizumab treatment rapidly improves symptoms and in some cases achieves complete disease remission. In systemic mastocytosis, omalizumab also improves symptoms and its prophylactic use to prevent anaphylactic reactions has also been discussed. In other pathologies such as atopic dermatitis, food allergy, allergic rhinitis, nasal polyposis, and keratoconjunctivitis, omalizumab significantly improves clinical manifestations. Omalizumab acts in two ways: by sequestering free IgE and by accelerating the dissociation of the IgE-Fc*ε* receptor I complex.

## 1. Introduction

IgE has unique properties among immunoglobulin isotypes and plays a central role in the pathophysiology of acute allergic reactions and chronic inflammatory allergic diseases. In genetically susceptible individuals, exposure to specific allergens results in an increase of specific IgE, which can bind onto effector cells through a high affinity receptor known as Fc*ε*RI expressed in mast cells and basophils [1].

IgE is very short-lived in plasma (about 1 day), but receptor-bound IgE can remain fixed to cells in tissues for weeks or months. Moreover, IgE binding to Fc*ε*RI increases cell survival and receptor upregulation [2, 3] and upon contact with a specific allergen induces the release of pharmacologically active mediators stored in the granules of mast cells (MC) and blood basophils (BS), resulting in clinical manifestations of type 1 hypersensitivity. In type 1 hypersensitivity, in the initial phase, an antigen (the allergen) is presented to antigen-specific CD4^+^ Th2 cells, which stimulate B-cell production of IgE antibodies that are also antigen-specific. During sensitization, the IgE antibodies bind to Fc*ε*RI on the surface of tissue MC and blood BS. Later exposure to the same allergen cross-links the bound IgE on sensitized cells, resulting in degranulation and secretion of preformed pharmacologically active mediators such as histamine. All of this occurs as an immediate reaction, starting within seconds. A late reaction caused by the induced synthesis and release of leukotrienes, chemokines, and cytokines by the activated mast cells allows the recruitment of other leukocytes, eosinophils, basophils, and Th2 lymphocytes to the site of inflammation. The allergic reaction includes symptoms like cough, bronchospasm, wheezing, diarrhea, and urticaria due to this process [1, 4].

IgE-mediated chronic diseases have classically been treated with antihistamines, corticoids, and other anti-inflammatory medications, but a number of patients do not respond to these treatments. The discovery and characterization of the pathways that drive different asthma phenotypes and our growing understanding of the pathophysiology of chronic urticaria (CU) have opened up new avenues for their treatment. To target the IgE with biological drugs has been pursued in the treatment of more severe cases of these pathologies.

The use of omalizumab (OmAb), an anti-IgE drug, is approved in severe allergic asthma not controlled by conventional treatment and in CU [5, 6]. IgE is known to be involved in other pathologies, and for this reason omalizumab is currently being assessed in conditions such as allergic rhinitis, atopic dermatitis, food allergies, mastocytosis, and eosinophilic gastrointestinal disease [7].

At present, the most interesting feature of omalizumab is its efficacy in conditions in which no successful treatment has previously been reported [5]. Perhaps unexpectedly, some reports have noted the drug's beneficial role in conditions, which apparently are not IgE-mediated [8]. If this efficacy is demonstrated, the uses of this biological drug may be extended to the treatment of other diseases.

In recent years, a number of other anti-IgE drugs have been developed. They will be discussed in this review, but it is by no means clear whether they will eventually be used in humans.

## 2. IgE-Related Chronic Diseases

### 2.1. Asthma

#### 2.1.1. Clinical Manifestations and Epidemiology

Asthma is a chronic inflammatory disease of the airways characterized by intermittent chest symptoms, variable airway obstruction, and bronchial hyperresponsiveness. In recent years, there has been a shift in the conception of asthma, which is no longer seen as a single disease but as a chronic condition with marked clinical heterogeneity over time [9]. Today, asthma is considered a complex syndrome with different phenotypes that share similar clinical manifestations but probably have different etiologies. The circumstances in which the symptoms appear are important because they can explain whether the condition is related to exposure to cold air, to pollen, or to other stimuli. Asthma symptoms occur paroxistically; that is, the patient is healthy for long periods although in severe cases the clinical manifestations persist. Various cells and inflammatory mediators are involved in this pathogenic process, which is conditioned partially by genetic factors [10]. Approximately 300 million people worldwide currently have asthma. In childhood, this disease is more frequent in males, but, in puberty, both sexes are affected equally and, in adulthood, it is more common in women. Mortality is around 180,000 deaths every year [11].

Classically, asthma has been divided into extrinsic and intrinsic phenotypes. Extrinsic asthma is characterized by hypersensitivity to a foreign molecule (substances, proteins) and is always associated with allergy. Intrinsic asthma covers all cases of asthma not attributable to allergies, such as asthma caused by sinus infections, chronic sinusitis, nasal polyps, acute bronchitis, colds, stress, or exercise. The attempts to understand the complexity of asthma presentation and the emergence of biological agents have led to a renewed interest in identifying clinically significant phenotypes. Currently, the study of asthma phenotypes is evolving, with a growing focus on their genetic base and corresponding biomarkers; but rather than creating an ever-expanding list of specific phenotypes, future research is likely to center on dissecting out the clinically relevant ones. The elucidation of asthma phenotypes has been further refined by the study of endotypes, which has provided information on the pathophysiological mechanisms present in different phenotypes [9, 12].

#### 2.1.2. Pathophysiology

Asthma has largely been viewed as a Th2-mediated process strongly linked to atopy and eosinophilic inflammation. However, a significant proportion of asthma cases do not present an increase in Th2 cytokines [13]. Non-Th2-mediated asthma is not as well understood as Th2-mediated asthma. In this review, we focus on Th2-mediated asthma, in which the role of IgE is well known.

Phenotyping studies have identified an early-onset allergic Th2-asthma phenotype (usually during preadolescence) and several late-onset Th2-related phenotypes (often at the age of 20 or later). The clinical phenotype of exercise-induced asthma (EIA) is also likely to have a Th2 component, given its eosinophil- and mast cell-related profile [14].

**Figure 1:**
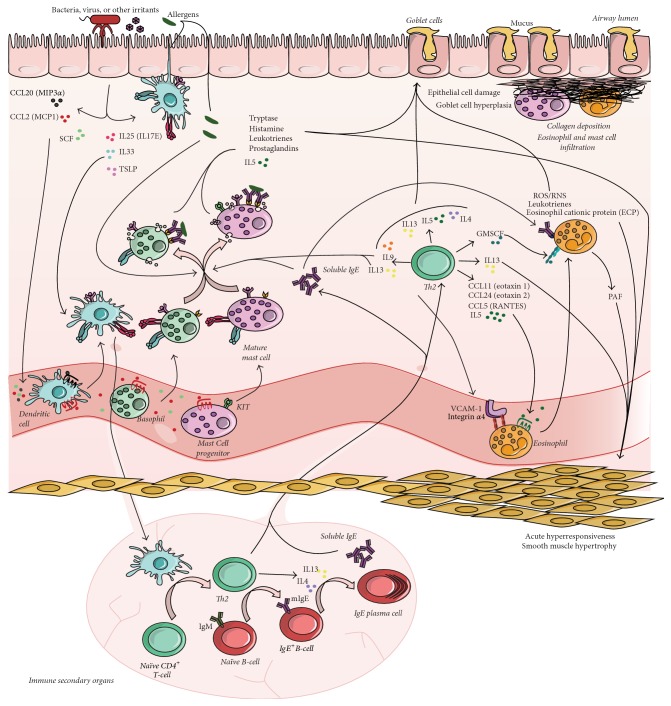
Pathophysiology of allergic asthma. Volatile allergens and/or other irritants can activate sentinel dendritic cells and/or epithelial cells in the airway epithelium that will also recruit and activate dendritic cells. The activation of dendritic cells will trigger Th2 responses, leading to the accumulation of soluble IgE as well as several cytokines. These cytokines will recruit mast cell progenitors, as well as basophils and eosinophils, which will be activated and secrete proinflammatory cytokines and other soluble factors such as histamine, tryptase, prostaglandins, and leukotrienes. As a consequence, there will be an increase in mucus production and bronchoconstriction (for mechanistic details, see text). If this activation is maintained, the airway will suffer persistent structural changes that will cause chronic bronchoconstriction due to fibrosis in the subepithelium and smooth muscle hypertrophy. mIgE: membrane IgE; PAF: platelet-activating factor.

Early-onset allergic Th2 asthma is the most studied phenotype, accounting for 50% of subjects with asthma, and it is linked with other allergic diseases such as allergic rhinitis and atopic dermatitis. The impairment it causes ranges from mild to severe [15]. This phenotype is associated with an increase in total and specific IgE [16]. There appears to be a genetic component to early-onset asthma, as evidenced by the family history of asthma in this group [17]. In allergic asthma, the allergen can directly activate sentinel dendritic cells (DC) present in the airway epithelium [18, 19] (Figure 1). However, bacterial epitopes or other injuries caused by virus or pollutants can act as initiators, as they can activate airway epithelial cells. These cells secrete several cytokines such as thymic stromal lymphopoietin (TSLP), IL25, and IL33, which can directly activate DC [20–22], and chemokines such as monocyte chemoattractant protein-1 (MCP1/also called CCL2) and macrophage inflammatory protein-3 (MIP3a/also called CCL20), which recruit basophils (BS). This also causes an increase in mast cells (MC) in the area [23] but it is not clear whether this is due to recruitment of MC progenitors, mainly by stem cell factor (SCF), or to proliferation of resident MC. DC then migrate to secondary immune organs and, via major histocompatibility complex class II (MHCII) and OXO40L, activate GATA3 transcription by naïve T-cells. The resulting Th2 cells will promote IgG to IgE switching of B-cells. In germinal centers, IL4 and IL13 cytokines, secreted by Th2 cells, cause IgE^+^ B-cells to become IgE plasma cells and to secrete soluble IgE against allergens. Soluble IgE, together with Th2 cells, return to the pulmonary tissue [24]. These IgE bind to Fc*ε*RI on the MC and BS cell surface. IgE-allergen complex promotes MC and BS degranulation of preformed mediators (histamine, tryptase, etc.) and secretion of* de novo* soluble components, including leukotrienes, prostaglandins, and other Th2 cytokines, which contribute to the prolonged inflammation and to the recruitment of more immune cells [25, 26]. Th2 cell-secreted IL9 cytokine contributes to this MC and BS activation [27].

Secretion of IL4 by Th2 cells promotes ICAM-1 and VCAM-1 expression on the surface of blood vessels [28], which allows eosinophils to attach and to be recruited, attracted by the action of eosinophil-attractant chemokines secreted by MC, BS, and Th2 cells such as IL5, eotaxin 1 and eotaxin 2, or RANTES, which also increase BS and MC recruitment and proliferation [29, 30]. All these cytokines and soluble molecules secreted by T-cells, MC, BS, and eosinophils cause inflammation, increase in goblet cell-produced mucus, and bronchoconstriction characteristic of an acute exacerbation. However, if this situation persists, these substances cause permanent epithelial damage and lead to structural changes, known as airway remodeling. This airway remodeling is marked by subepithelial fibrosis, due to an increase in the epithelial-mesenchymal trophic unit and collagen deposition characterized by eosinophil and mast cell infiltration, as well as smooth muscle hypertrophy, leading to chronic bronchoconstriction and reduced airway responses to bronchodilators [31] (Figure 1).

Late-onset Th2 asthma is characterized by marked eosinophilia, less atopy, and recurrent exacerbations. This form of asthma is believed to be unrelated to allergic triggers. A family history of this asthma is also less commonly observed and the genetics of this phenotype have not been specifically studied. The lack of clinical allergy in this phenotype suggests that the Th2 process differs from and is probably more complex than the early-onset allergic phenotype [13]. Aspirin-exacerbated respiratory disease (AERD) is a subphenotype of persistent eosinophilic asthma and is widely believed to be an endotype. It comprises a type of adult-onset, highly eosinophilic asthma with inflammation of nasal and bronchial tissues and non-IgE-mediated response to aspirin or other cyclooxygenase-1 inhibitors. AERD pathophysiology is characterized by increased cysteinyl leukotriene production [32].

Furthermore, numerous environmental factors such as smoking, hormonal changes, infections, and obesity are comorbidities and confounders that can alter asthma phenotypes and influence the underlying immunoinflammatory process.

### 2.2. Chronic Urticaria

#### 2.2.1. Clinical Manifestations, Classification, and Epidemiology

Urticaria is characterized by pruritic wheals that develop quickly with a central edema and a surrounding area of erythema. The size of the wheals is variable and the lesions last from one to 24 hours. The disease may be accompanied by angioedema, defined as cutaneous or mucosal swelling that is generally nonpruritic but is painful and lasts from one to three days [6]. Urticaria can be divided into two groups on the basis of its clinical manifestations: the acute form, which lasts less than six weeks and is often allergic, and chronic spontaneous urticaria (CSU), also known as chronic spontaneous/idiopathic urticaria, which presents daily or almost daily wheals for more than six weeks. This condition affects 0.1%–0.8% of the population [33, 34]. It includes a subpopulation of patients with positive autoimmune serology (up to 30%) to the IgE receptor, IgE, and antithyroid antibodies [35]. The persistence and severity of the symptoms correlate with positive autoimmune serology, more intense inflammation in skin biopsy, and resistance to antihistamines [36]. A third general form of the condition is known as inducible urticaria (physical, cold, cholinergic urticaria, or dermatographism) but will not be addressed here.

#### 2.2.2. Pathophysiology

Chronic spontaneous urticaria may occur as a result of mast cell and basophil release of bioactive mediators. However, the mechanism of mast cell degranulation in urticaria patients remains unclear. Currently, we know that immunological and nonimmunological factors are involved. The key role in the pathogenesis of CSU is played by the vasoactive mediators released from dermal mast cells. Histamine is the most prominent of these mediators although there are others such as eicosanoids, cytokines, and proteases. Histamine acts on H1 receptors (85%) and on H2 (15%) in the skin. Histamine binding to H1 receptors provokes pruritus, vasodilatation, and edema [37]. Mechanisms other than histamine release which have been implicated in CSU include autoimmunity and abnormalities in basophil signal transduction and basopenia [37].

**Figure 2:**
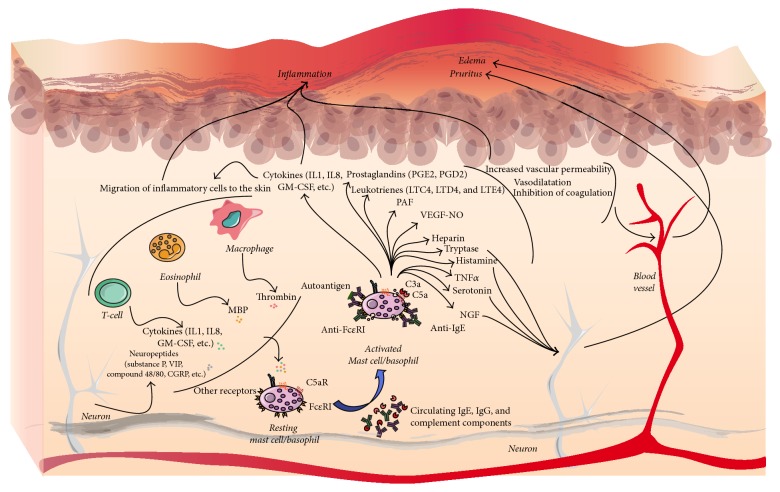
Pathophysiology of urticaria. In CSU patients, stimulation of mast cells (MC) and basophils (BS) can be triggered by IgE against autoantigens, by IgG against Fc*ε*RI or IgE against IgE itself, or by complement. Moreover, MC and BS can be stimulated by molecules secreted by other immune cells or neurons. Once activated, MC and BS secrete several preformed mediators, such as histamine or tryptase, and other* de novo* mediators, such as prostaglandins and leukotrienes, will promote inflammation, vascular permeability, and vasodilatation, as well as neuron stimulation. These effects are transduced into edema and pruritus. Secretion of cytokines by MC and BS triggers migration of other immune cells to the skin, which will contribute to skin inflammation. MBP: major basic protein; NFG: nerve growth factor; PAF: platelet-activating factor; NO: nitric oxide.

In terms of pathophysiology, three categories of CSU have been defined (Figure 2). 

(*1) Allergic*. In this case, an allergen acts by stimulating the production of IgE, which binds to the Fc*ε*RI, leading to mast cell and basophil degranulation. 

(*2) Autoimmune*. An autoimmune etiology is suggested by several findings. Autologous intradermal injection of sera from patients with CSU causes wheal and flare reactions [38]. Moreover, the analysis of urticaria patients' sera reveals IgG autoantibodies to the alpha subunit of Fc*ε*RI or to IgE itself [39]. IgG autoantibodies against IgE or the IgE high affinity receptor are produced in almost half of the patients with CSU. The autoantibody cross-linking against the alpha subunit of Fc*ε*RI induces degranulation of the mast cells and blood basophils, which is followed by the release of histamine [40]. IgG_1_ and IgG_3_ are the main anti-Fc*ε*RI autoantibody subclasses found in CSU [41]. The role of complement has been demonstrated, since the presence of C5a increases the histamine released by anti-Fc*ε*RI autoantibodies in normal human mast cells and basophils* in vitro* [42].

Furthermore, a small percentage of blood basophils, histamine-releasing autoantibodies, and HLA-DR alleles that are generally associated with autoimmune diseases are frequently increased in CSU [43]. 

(*3) Nonimmunological*. The mechanisms are independent of IgE and Fc*ε*RI. This group includes inducible urticaria and urticaria secondary to drugs. Moreover, other CSU patients without autoimmunity or increased serum IgE could also be included: in these cases, the trigger is not known, but it may involve alterations in other unknown molecular mechanisms.

## 3. Anti-IgE-Based Treatments

Since the identification of IgE as major stimuli in the inflammatory cascade, the development of agents to target IgE has thrived. Among them, the anti-IgE biological omalizumab has been one of the most successful.

### 3.1. Anti-IgE Drug Omalizumab: Mechanism of Action

Omalizumab (OmAb) is a recombinant humanized monoclonal antibody that was designed to bind to IgE on the Fc (constant fragment) portion, C epsilon 3 locus, in the same domain where IgE is bound to Fc*ε*RI [44, 45]. This drug was synthetized with the aim of sequestering free IgE and reducing allergic inflammation [5]. The drug is administered subcutaneously and is absorbed slowly. The peak of serum concentration is reached after 7-8 days [5] and it is eliminated via the reticuloendothelial system, having a half-life of around 26 days.

**Figure 3:**
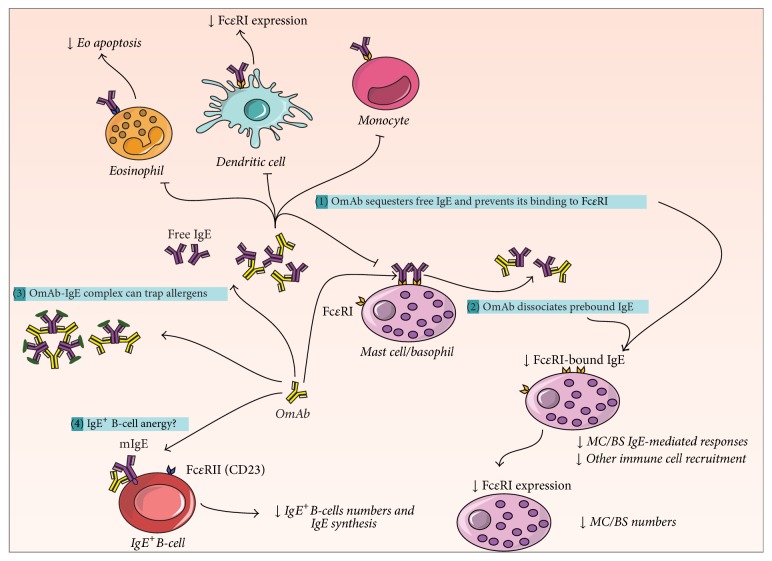
Mechanism of action of OmAb. The two main described mechanisms of action of OmAb are (1) its ability to sequester free IgE and block its binding to IgE receptors (Fc*ε*RI) and (2) its ability to accelerate the dissociation of IgE bound to Fc*ε*RI in mast cells (MC) and basophils (BS). As a consequence, there is a reduction of IgE-triggered responses, as well as a reduction of the number of eosinophils (Eo), mast cells (MC), and basophils (BS). As a complementary mechanism, IgE complexed with OmAb may trap allergens (3). Another less understood mechanism would lead to a reduction of IgE^+^ B-cell numbers and a decrease of IgE synthesis (4). mIgE: membrane IgE.

It has been accepted for a long time that OmAb acts on the free IgE (mechanism (1) in Figure 3) and may abolish the binding of IgE to Fc*ε*RI^+^ or Fc*ε*RII^+^ (CD23) cells, B-cells, dendritic cells (DC), eosinophils (Eo), and monocytes. Interestingly, in recent years, the drug's action has been shown to go further, dissociating bound IgE from the IgE-Fc*ε*RI complex (mechanism (2) in Figure 3) [46, 47]. Thus, at a physiological concentration range, OmAb may accelerate the dissociation of the preformed IgE-Fc*ε*RI complex on the surfaces of mast cells and basophils in addition to its ability to neutralize the free IgE, leading to an impairment of the IgE-inflammatory signaling cascade [47]. Moreover, the density of Fc*ε*RI expression on basophils, mast cells, and dendritic cells falls notably in patients receiving anti-IgE treatment within the first week of OmAb application [8]. This may be because the IgE stabilizes the receptor on the cell surface and prevents its internalization; thus, a reduction of the immunoglobulin leads to a decrease in receptor expression [48–50]. All these events make these cells unresponsive to IgE triggering and reduce symptoms such as inflammation, edema, and pruritus. Finally, this leads to a reduction in MC/BS numbers. Interestingly, the reduction in Fc*ε*RI expression has also been demonstrated in dendritic cells [51].

Likewise, as a complementary mechanism, it has been proposed that OmAb-IgE complexes can bind to antigens and act as competitive inhibitors (mechanism (3) described in Figure 3) [52].

It has also been published that OmAb may target membrane-IgE (mIgE) in IgE^+^ B-cells, reducing IL4R expression and IgE synthesis and decreasing the number of these cells, possibly by causing B-cell anergy [53] (mechanism (4) described in Figure 3). OmAb has also been reported to cause eosinophil apoptosis [54], a finding that is in agreement with the fall in blood eosinophilia found in asthma patients after OmAb administration [55]. However, it is not clear whether this is due to a direct effect of OmAb or is caused by the reduction of IgE or the reduced secretion of cytokines by T-cells.

To sum up, OmAb may act via several mechanisms, which appear to affect not only IgE-triggered events, but also the viability of the different cells, involved in these pathologies. This leads to a rapid and prolonged reduction of the symptomatology.

### 3.2. Omalizumab in Asthma

While most asthma is controlled with anti-inflammatory and bronchodilator medications irrespective of phenotype, a minority of patients, around 10%, respond poorly. Thus, the definition of “severe” asthma is applied to patients whose symptoms or exacerbations require the use of a high-dose inhaled corticosteroid plus a second controller, or whose disease persists in spite of treatment [56, 57]. The understanding of asthma physiopathology has allowed the design of treatments for these persistent cases based on anti-IgE monoclonal antibodies such as OmAb.

Many clinical studies have reported the effectiveness of OmAb and the use of the drug has been extensively reviewed in the literature. Among the largest studies, a systematic study in 2006 analyzed its efficacy in allergic asthma, based on data obtained from 14 studies including a total of 3,143 patients. The results showed that inhaled corticosteroid therapy, which is the mainstay of asthma therapy, was reduced by more than 50% in a significant number of patients after OmAb treatment, and some patients were able to discontinue inhaled corticosteroid therapy entirely [58]. Other clinical trials carried out in recent years have confirmed that OmAb treatment improves symptoms and reduces the frequency of asthma exacerbations and the need for high doses of inhaled corticosteroids [8].

In several real-life studies, the use of omalizumab has been associated with an absence of exacerbations and an improvement in quality of life, which is reflected in reduced hospital admissions and emergency visits [8].

In an attempt to elucidate the drug's mechanism of action, another placebo-controlled trial was conducted in 41 adult patients with severe, nonatopic refractory asthma. Interestingly, OmAb regulated Fc*ε*RI expression negatively on basophils and plasmacytoid dendritic cells and increased forced expiratory volume in the first minute (FEV1) compared with baseline after 16 weeks in patients with severe nonatopic asthma, as it does in severe atopic asthma [59]. This finding points to a possible role of IgE in nonatopic asthma.

The standard duration of treatment with OmAb has not been established to date. A follow-up study showed that, after six years of OmAb treatment, most patients had mild and stable asthma in the ensuing three years after treatment discontinuation [60]. It has been suggested that the persistence of the effects of OmAb may be due to its ability to curtail airway remodeling in patients with asthma. In fact, it has been found that OmAb significantly decreased the airway wall area, the percentage of wall area, and the luminal area of the right apical bronchial segments, whereas no change was achieved with conventional therapy [61]. After one year of OmAb treatment, a significant mean reduction in eosinophilic infiltration was recorded as well as a reduction in reticular base membrane in bronchial biopsies from patients with severe persistent allergic asthma. These findings indicate that OmAb may modify the course of the disease due to their possible influence curtailing airway remodeling.

### 3.3. Omalizumab in Chronic Urticaria

Nowadays, several options are available for treating chronic urticaria. Practical measures include the avoidance of aggravating factors such as drugs (nonsteroidal anti-inflammatory drugs, NSAIDs), alcohol, stress, and local heat and friction. Currently, the guidelines recommend a stepwise approach, which includes nonsedating antihistamines as a first line of treatment or combinations with oral corticosteroids. Control is often insufficient and additional therapies have included leukotriene antagonists or cyclosporine, and more recently OmAb has been added if symptoms persist [6].

The use of OmAb in urticaria has focused mainly on CSU with autoimmune form [62]. The effect of OmAb on CSU with or without angioedema has been demonstrated in several double-blinded randomized placebo-controlled studies including almost 1200 patients, with relatively few side effects [63–66]. Although the OmAb dose for CSU is set at 300 mg every 4 weeks, a dose of 150 mg every 4 weeks also achieves an effect in some patients, and in other cases the dose needs to be increased to 300 mg every two weeks. In some cases, signs and symptoms of urticaria cease after a few days of treatment, a faster effect than in asthma, which begins to present improvement after a week at the earliest [62]. These findings suggested another mechanism of action for OmAb, apart from its ability to sequester free IgE. Lowering free IgE levels may downregulate the levels of IgE receptor expression density on the surface of mast cells in the long term, but this would not appear to be responsible for the rapid improvement reported in the clinical symptoms; a more likely reason is OmAb's ability to dissociate prebound IgE from Fc*Ɛ*RI [46, 47]. Nevertheless, it is still unclear how OmAb works in CSU: in addition, the fact that OmAb is not effective in all patients suggests the involvement of mechanisms/pathways in CSU other than the IgE cascade.

Interestingly, OmAb has also been reported to be effective in treating other forms of urticaria: cold urticaria, solar urticaria, cholinergic urticaria, delayed pressure, and symptomatic urticaria factitia, although the role of IgE in these urticarial conditions is unknown [67–71].

### 3.4. Off-Label Use of Omalizumab in Other Diseases

#### 3.4.1. Systemic Mastocytosis, Hyperimmunoglobulin E Syndrome, and Eosinophilic Gastroenteritis

Mastocytosis is a heterogeneous disorder that results from abnormal proliferation and accumulation of mast cells in one or more organs. When this infiltration extends to extracutaneous organs such as bone marrow, liver, spleen, and gastrointestinal tract in spite of the cutaneous infiltration, systemic mastocytosis is diagnosed. Increased local concentration of soluble mast cell growth factors in lesions is believed to stimulate mast cell proliferation. Impaired mast cell apoptosis and interleukin-6 have also been implicated, as evidenced by BCL-2 upregulation and high IL6 levels in tissue. Most patients with the systemic form present an activating point mutation in the c-kit gene in codon 816 (D816V), which is thought to contribute to the abnormal proliferation of mast cells and enhanced mast cell survival [72]. OmAb has been reported to be safe and effective in preventing recurrent anaphylaxis [73]. Since OmAb reduces the expression of Fc*ε*RI on circulating basophils and mast cells, it seems to lower their activity and thus reduces their potential reactivity [74, 75]. Curiously, there is no evidence of the capacity of OmAb to decrease mast cell numbers, because the serum tryptase levels in several patients with mastocytosis do not vary during the period of response [76]. In another study, serum tryptase was reported to decrease during OmAb therapy in two mastocytosis patients, but it remained unchanged in two others [77]. The mechanisms underlying the symptomatic improvement in patients with systemic mastocytosis treated with OmAb are still not fully understood.

Hyperimmunoglobulin E syndrome (HIES) is a heterogeneous group of immune disorders characterized by very high levels of serum IgE, dermatitis, and recurrent skin and lung infections. There are two forms of HIES: a dominant form caused by mutations in STAT3 and a recessive form for which a genetic cause is unclear. These syndromes have distinct presentations, courses, and outcomes but both present clear increases in IgE levels. Studies report clinical improvements in patients with high serum IgE levels and presenting severe atopic eczema and in patients presenting several other symptoms after OmAb treatment [78, 79].

Eosinophilic gastroenteritis is characterized by patchy or diffuse eosinophilic infiltration of any part of the gastrointestinal tract. Anti-IgE treatment with OmAb is associated with a 35–45% drop in peripheral blood eosinophil count and decreases in duodenal and antral eosinophils. It effectively blocks CD23-mediated allergen binding to B-cells [80].

#### 3.4.2. Allergic Rhinitis, Nasal Polyposis, and IgE-Related Respiratory Diseases

There is a close relationship between asthma and allergic rhinitis. For this reason, OmAb was expected to be effective in the treatment of concomitant rhinitis in patients with asthma. Indeed, in one trial, the odds ratio for a positive effect on rhinitis was 3.56, indicating that the probability of improvement was three and a half times higher in subjects treated with OmAb [81]. In a 2002 double-blinded, randomized trial, combination therapy of OmAb with specific immunotherapy (SIT) for birch and pollen reduced symptom load over two pollen seasons by 48% compared with SIT alone [82].

In nasal polyposis, the results of using OmAb are less obvious since this condition appears in nonallergic patients. Significantly, high levels of IgE in polyps have been related to* Staphylococcus aureus* enterotoxin acting as a superantigen rather than atopy. Interestingly, IgE antibodies against* S. aureus* enterotoxin were found in a significant higher concentration in severe asthma patients compared to controls suggesting a relationship between these antibodies and asthma severity [83]. In this context, several studies with OmAb have also demonstrated clinical efficacy in the treatment of nasal polyps with comorbid asthma [84, 85].

Omalizumab has also demonstrated its clinical relevance in patients with allergic bronchopulmonary aspergillosis (ABPA), an allergic reaction to* Aspergillus* characterized by high IgE levels, which usually occurs in combination with cystic fibrosis (CF) [86, 87]. Several case series have also reported success with OmAb in ABPA patients without CF [88, 89].

#### 3.4.3. Atopic Dermatitis and Bullous Pemphigoid

Atopic dermatitis (AD) is one of the most frequent chronic inflammatory skin disorders associated with elevated serum IgE levels. Acute AD skin lesions are characterized by intensely pruritic, erythematous papules associated with epidermal intercellular edema as well as increased Langerhans cells, inflammatory dendritic epidermal cells, macrophages, eosinophils, and activated CD4-positive Th2 cells. The results with the use of OmAb for atopic dermatitis are controversial: several case reports investigating anti-IgE therapy in patients with AD have found symptomatic improvement [90, 91], but others report negative responses in patients with severe AD treated with a four-month course of OmAb [92], or a favorable response in only 6 of 11 patients [78]. More randomized controlled trials including a placebo control group are needed to validate the effectiveness of OmAb for AD.

Bullous pemphigoid (BP) is an acquired autoimmune disease presenting subepidermal blistering, eosinophilia, and severe itching. It is characterized by the presence of autoantibodies against the 230 kDa bullous pemphigoid antigen within basal keratinocytes and the 180 kDa type XVII collagen in the basement membrane zone lying between the epidermis and dermis. IgE-specific antibodies against type XVII collagen were detected in sera and biopsy samples from the majority of BP patients and these IgE autoantibodies have been shown to be pathogenic. Clinical trials with OmAb showed effectiveness in several cases [93, 94].

#### 3.4.4. Food Allergy and Food-Related Anaphylaxis

OmAb induced a significant increase in the threshold dose for an oral food challenge with peanuts causing allergic symptoms [95]. OmAb has also been useful in introducing oral immunotherapy (OIT) in food-allergic patients. In a pilot study with children with clinical reactions to cow's milk, OmAb treatment combined with oral milk desensitization permitted rapid milk dose escalation in the majority of subjects. [96]. OmAb was also reported to be effective in tolerability of various food allergies during an OIT protocol in 25 patients [97]. Thus, OmAb in combination with oral desensitization has a potential value for the treatment of food allergy.

#### 3.4.5. Atopic Keratoconjunctivitis

Atopic keratoconjunctivitis is a severe ocular disorder of the cornea with immediate and delayed hypersensitivity reactions, which can produce loss of visual acuity and blindness. In an open-label trial, six patients treated with OmAb showed an improvement in their ocular symptoms [98].

### 3.5. Other Anti-IgE-Based Therapies

#### 3.5.1. C*ε*mX Monoclonal Antibodies

Membrane-bound IgE (mIgE) is part of the IgE-BCR and is essential for generating isotype-specific IgE responses. On mIgE^+^ B-cells, the membrane-bound *ε*-chain exists predominantly in the long isoform, thus providing an attractive site for immunologic targeting of mIgE^+^ cells. C*ε*mX-specific antibodies have proved potentially useful for targeting mIgE^+^ cells to control IgE production [99]. These were the bases for the production of quilizumab, a humanized IgG1 monoclonal antibody that binds to the M1-prime segment present only on mIgE, but not on soluble IgE in serum. In phase I and II studies, quilizumab reduced serum total IgE by approximately 25%. These decreases were sustained for at least six months after the last dose, in contrast to OmAb, which must be administered every 2–4 weeks to maintain reduced IgE levels [100]. Unfortunately, in adults with uncontrolled allergic asthma, a 36-week treatment with quilizumab did not have a clinically significant impact on exacerbation rate, lung function, or quality of life [101]. Nor did its use in patients with refractory CSU achieve a clinically significant improvement, although it reduced median serum IgE by approximately 30% [102].

#### 3.5.2. Ligelizumab

Ligelizumab (QGE031) is a humanized IgG1 monoclonal antibody that binds with higher affinity to the C epsilon 3 domain of IgE. Designed to achieve greater IgE suppression than OmAb, it may overcome some of the limitations associated with OmAb dosing and achieve better clinical outcomes. Data from preclinical experiments and two phase I randomized, double-blind, placebo-controlled clinical trials showed QGE031 to be superior to OmAb in suppressing free IgE and basophil surface expression of Fc*ε*RI and IgE. These effects allowed almost complete suppression of the skin prick response to the allergen, which was superior in extent and duration compared to the case with OmAb [103].

#### 3.5.3. Bispecific Antibodies and Designed Ankyrin Repeat Proteins (DARPins)

Some studies have shown that the use of bispecific antibodies that cross-link Fc*ε*RI and the low-affinity IgG receptor (Fc*γ*RIIb) on mast cells and basophils inhibits allergen-induced cell degranulation [104, 105].

DARPins are genetically engineered proteins that typically exhibit highly specific and high affinity target protein binding. One study reported a specific anti-IgE DARPin (DE53-Fc) fused to the Fc part of a human IgG1, which inhibited allergen-induced basophil activation in samples of different donors via Fc*γ*RIIb [106]. For its part, DARPin E2_79 is able to block IgE:Fc*ε*RI interactions and actively stimulates the dissociation of preformed ligand-receptor complexes [107].

Bispecific antibodies and DARPins represent promising drug candidates for the treatment of IgE-related diseases but their potential for use in humans is still to be confirmed.

#### 3.5.4. IgE-R419N-Fc3-4

Structural studies of OmAb have facilitated the design of an IgE-Fc3-4 mutant (IgE-R419N-Fc3-4) that is resistant to OmAb neutralization but is able to bind Fc*ε*RI and Fc*ε*RII. The IgE-R419N-Fc3-4 mutant, in combination with OmAb, can effectively exchange cell-bound IgE for IgE-R419N-Fc3-4 and this dual inhibitor treatment blocks basophil activation more powerfully than either inhibitor alone. This approach, involving simultaneous depletion of antigen-specific IgE while engaging Fc*ε*RI and Fc*ε*RII receptors with an IgE variant, can be used to further test the role of IgE-dependent regulatory pathways during anti-IgE treatment and may provide a promising way forward for enhancing current anti-IgE therapies [108].

## 4. Conclusion

A better understanding of asthma and chronic urticaria phenotypes and endotypes will allow us to select treatments based on the likelihood of response, thereby improving the control and quality of life of these patients. The number of biological treatments for these diseases continues to grow.

Targeting IgE has proved to be a successful approach to IgE-related diseases with poor response to traditional treatments. Although the indications for OmAb are currently limited to allergic asthma and CSU, its potential in the treatment of other allergic comorbidities is becoming increasingly clear. In fact, the off-label uses of OmAb have shown promising results in a variety of diseases in which IgE has a limited or unconfirmed role. The knowledge of OmAb's mechanism of action may help to elucidate the relationships between all the factors interacting in IgE-mediated pathologies and may shed some light on some others that are not IgE-mediated.

Thus, more studies are needed to uncover the molecular insights of these pathologies and the mechanism of action of anti-IgE biological drugs such as OmAb and to test the efficacy of new IgE-targeted drugs in humans.
